# Urinary Metabolites and Survival in Malignant Mesothelioma

**DOI:** 10.1101/2025.09.23.25336488

**Published:** 2025-09-25

**Authors:** Leila Toulabi, Joshua K. Stone, Mohammed Khan, Daxesh P. Patel, Raffit Hassan, Curtis C. Harris

**Affiliations:** a Laboratory of Human Carcinogenesis, Center for Cancer Research, National Cancer Institute, Bethesda, Maryland, USA; b Thoracic and GI Malignancies Branch, Center for Cancer Research, National Cancer Institute, Bethesda, Maryland, USA.

**Keywords:** biomarker, Mesothelioma, metabolomics, urine, metabolomics, prognosis, response to therapy

## Abstract

**Background:**

Malignant mesothelioma is a rare and aggressive malignancy with limited treatment options and poor survival outcomes. Current biomarkers such as MESOMARK, fibulin-3, and osteopontin show diagnostic potential but remain hampered by inconsistent sensitivity and reproducibility. Urine-based metabolites may provide a simple, noninvasive alternative for prognosis and disease monitoring.

**Methods:**

We evaluated whether four urinary metabolites—creatine riboside, N-acetylneuraminic acid, cortisol sulfate, and cholestane pentol—previously associated with prognosis in lung and liver cancer, are prognostic in mesothelioma. Urinary metabolite concentrations were quantified in a clinically annotated cohort with survival follow-up. Associations with overall survival and a validated poor-prognostic gene expression signature were analyzed.

**Results:**

Elevated urinary concentrations of the four metabolites were significantly correlated with poor-prognostic gene expression profiles. Patient survival declined progressively with the number of elevated metabolites, with the poorest outcomes observed in individuals harboring three or more high metabolites. These associations were independent of clinical variables and consistent in sensitivity analyses, suggesting that metabolite elevations reflect aggressive tumor biology.

**Interpretation:**

Urinary metabolites represent promising prognostic biomarkers in mesothelioma. Their additive and dose-dependent relationship with survival highlights their potential clinical utility for risk stratification, patient monitoring, and trial design. Because urine collection is noninvasive and repeatable, these biomarkers could complement existing blood-based assays and provide an accessible tool for clinical practice. Larger, multi-institutional studies are warranted to validate their prognostic significance and enable translation into patient care.

## INTRODUCTION

Malignant mesothelioma is an uncommon but highly aggressive cancer that arises from the mesothelial lining of the pleura, peritoneum, or pericardium. The disease is most strongly associated with prior asbestos exposure and is characterized by a decades-long latency period, insidious onset, and rapid progression once clinically apparent. Despite advances in multimodal management—including surgery, chemotherapy, and immune checkpoint inhibition—the prognosis for mesothelioma remains poor, with median survival rarely exceeding 12 to 18 months.^1^ A major barrier to improving outcomes is the absence of reliable, clinically validated biomarkers that enable earlier detection, provide robust prognostic information, and support more precise patient stratification for treatment and clinical trials.

Currently available biomarkers are predominantly blood-based. The MESOMARK assay, which quantifies soluble mesothelin-related peptides, is the only FDA-cleared test for mesothelioma. While MESOMARK offers high specificity, its sensitivity is limited, especially for early-stage disease, which restricts its clinical utility.^2^ Other candidate markers, including fibulin-3 and osteopontin, have demonstrated greater sensitivity in certain contexts but face substantial challenges. Fibulin-3 levels vary markedly between plasma and effusion samples, impairing reproducibility, while both fibulin-3 and osteopontin are subject to thrombin cleavage in serum, which complicates measurement and interpretation.^3, 4^ Collectively, these limitations highlight the urgent need for complementary biomarkers that are reproducible across sample types, less prone to preanalytical variability, and ideally accessible through noninvasive collection.

Metabolomics has emerged as a powerful approach for biomarker discovery, offering unique advantages compared with proteomic or genomic assays. Metabolites represent the downstream products of cellular activity and capture integrated effects of genetic alterations, transcriptomic changes, host responses, and environmental exposures. Importantly, urinary metabolites can be readily and repeatedly measured through noninvasive sampling. Urine collection avoids many of the challenges associated with blood, such as clotting variability and processing artifacts, making it a particularly attractive medium for biomarker development. Previous studies in lung and liver cancer have identified four urinary metabolites—creatine riboside, N-acetylneuraminic acid, cortisol sulfate, and cholestane pentol—as robust prognostic indicators.^5, 6^

These metabolites implicate critical pathways in energy metabolism, sialic acid biology, steroid hormone regulation, and cholesterol metabolism, all of which are central to cancer progression and patient outcomes. Their reproducible association with poor prognosis in multiple tumor types suggests that they may reflect fundamental aspects of tumor biology that extend beyond organ-specific contexts.

We hypothesized that these metabolites would also be prognostic in mesothelioma, given the shared hallmarks of metabolic reprogramming, inflammation, and aggressive clinical behavior. To test this hypothesis, we analyzed urinary concentrations of the four metabolites in a clinically annotated mesothelioma cohort with long-term survival follow-up. Associations with overall survival and with a validated poor-prognostic gene expression signature were evaluated to determine whether urinary metabolite profiles could serve as practical and clinically relevant prognostic tools.

Here, we report that elevated levels of these urinary metabolites were strongly associated with adverse molecular profiles and worse survival outcomes in mesothelioma. Survival decreased progressively with the number of elevated metabolites, with patients harboring three or more high metabolites experiencing the poorest prognosis. These findings provide evidence that urinary metabolite profiling offers prognostic information complementary to existing blood-based assays. Because urine collection is simple, noninvasive, and inexpensive, these metabolites hold promise as translational biomarkers for risk stratification, longitudinal monitoring, and clinical trial enrichment in mesothelioma. Larger, multi-institutional studies are warranted to validate their clinical utility and accelerate their integration into practice.

## MATERIALS AND METHODS

Biospecimens from the Tissue Procurement and Natural History Study of Patients With Malignant Mesothelioma (NCT01950572) cohort were collected as per Institutional Review Board approved protocol and in agreement with the principles of the International Conference on Harmonisation - Good Clinical Practice (ICH-GCP) guidelines. All patients provided written informed consent. In this study 425 patients whom the diagnosis of mesothelioma in all patients was confirmed by Board-Certified Anatomic Pathologists of the NCI Laboratory of Pathology were enrolled between September 2013 and November 2019, out of RNA-seq data was available for 100 patients. Patients were enrolled regardless of asbestos exposure, gender, age at diagnosis, or personal or family history of cancer. This cohort We analyzed 95 urine samples from patients with available RNA-seq data enrolled in the Tissue Procurement and Natural History Study of Patients with Malignant Mesothelioma cohort. Urine samples from participants were gathered, frozen, and preserved at −80°C. We performed LC-MS/MS analysis on urine from 234 mesothelioma patients who participated in the clinical trial at the Thoracic and Gastrointestinal (GI) Malignancies Branch, CCR, NCI, NIH ^7^, and we focused on 95 of these patients for whom expression data was available (Table 1).

Urine extracts were prepared as described previously and analyzed by ultraperformance liquid chromatography MS/MS (UPLC-MS/MS) ^5^. Standards were either synthetic metabolites or purchased (MilliporeSigma, Cambridge Isotope) ^8^. Metabolite concentrations of creatine riboside (CR), N-acetylneuraminic acid (NANA), cortisol sulfate (CS), and 27-nor-5β-cholestane-3α,7α,12α,24,25 pentol (cholestane pentol, CP) were measured using a XEVO G2 ESI QTOF mass spectrometer. CR (mass to charge ratio (m/z) = 264.1196 and retention time (RT) = 0.4 min) and CP (m/z = 561.3435, RT = 6.3) were measured in electrospray ionization (ESI) positive mode. NANA (m/z = 308.0982, RT = 0.4 min) and CS (m/z 441.162, RT = 5.5) were measured in ESI negative mode. Concentrations were then calculated using calibration curves of analytical standard solutions (Masslynx, Waters) and then normalized to creatinine levels within each urine sample. Patient metabolite concentrations below the median were classified ‘low’ and those equal to or greater than the median were classified ‘high’ (median concentrations CR 1.987 μM, NANA 9.234 μM, CS 21.011 nM, CP 5.877 nM).

All statistical analyses were performed R (v4.1.1) using the Mann-Whitney *U* test. Kaplan-Meier survival analysis (log-rank test) was performed using the R packages survminer and survival and STATA. A *P* value of less than 0.05 was considered statistically significant.

## RESULTS

### Association of Urinary Metabolites with a Prognostic Gene Signature

**Figure 1.**
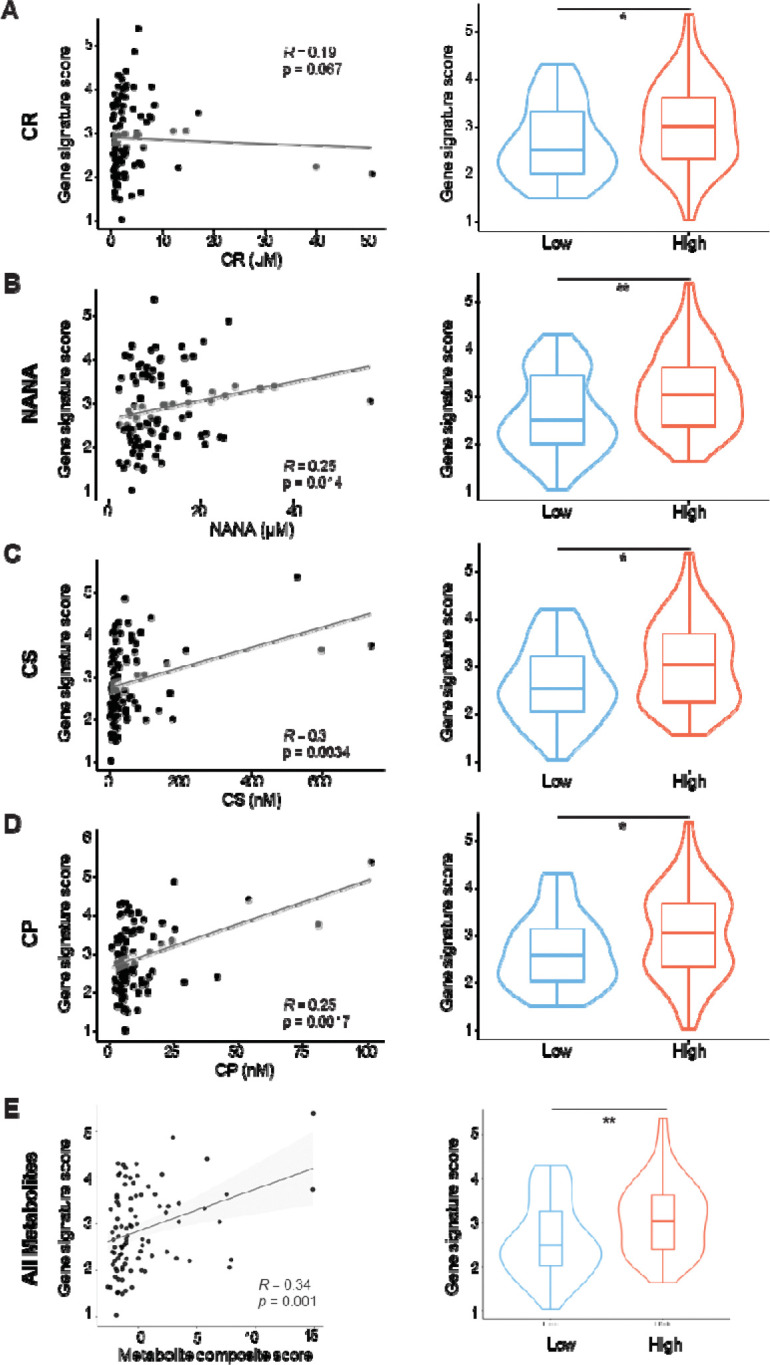
Mesothelioma 48-gene signature score is elevated in metabolite-high patients. (Left) Spearman correlation scatterplots between metabolite concentrations and 48-gene signature scores. (Right) Metabolite concentrations were individually classified as high (≥ median) or low (< median). (A) Creatine riboside, CR (low n = 48, high n = 47). (B) *N*-acetylneuraminic acid, NANA (low n = 48, high n = 47). (C) Cortisol sulfate, CS (low n = 46, high n = 47). (D) Cholestane pentol, CP (low n = 46, high n = 48). (E) All 4 metabolites (low n = 46, high n = 47). *p < 0.05, ** *p* < 0.01.

Urinary concentrations of creatine riboside (CR), N-acetylneuraminic acid (NANA), cortisol sulfate (CS), and cholestane pentol (CP) were evaluated in the Mesothelioma Natural History cohort (NCT01950572). All four metabolites were positively correlated with a validated 48-gene poor-prognostic expression signature.^7^ Patients classified as high for individual metabolites, or for multiple metabolites in combination, had significantly higher gene signature scores than those with low concentrations (Figure 1). These results demonstrate that metabolite elevation is concordant with molecular features of aggressive disease.

### Clinicodemographic Associations

We next assessed whether metabolite concentrations varied by demographic or clinical variables. No significant differences were observed between patients <60 vs ≥60 years (Figure S1) or between pleural and peritoneal disease sites (Figure S2). Similarly, no global sex-based differences were detected, although CP was modestly higher in females compared with males (Figure S3). Collectively, these analyses indicate that metabolite levels are largely independent of age, sex, or disease site, supporting their potential as robust, broadly applicable biomarkers.

### Prognostic Value for Survival

**Figure 2.**
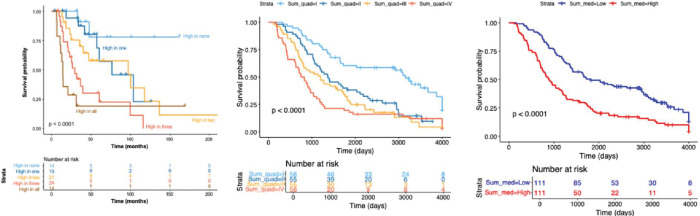
High urinary metabolite concentration is predictive of poor survival. **A)** Metabolites were individually classified as high (≥ median) or low (< median), then high/low classifiers were combined for each patient. **B)** KM plot for composite score by quartile value **C)** KM plot for composite score by median value. *p*-values were calculated using the log-rank statistic.

Kaplan–Meier analysis revealed that urinary metabolite levels were strongly associated with overall survival. Patients were classified according to the number of metabolites above the median concentration. Median survival decreased progressively as the number of elevated metabolites increased, with the poorest outcomes observed among patients with three or more high metabolites (log-rank p < .0001; Figure 2A). This graded relationship suggests an additive prognostic effect across metabolites.

### Composite Metabolite Score

To integrate information across the four biomarkers, we generated a standardized composite score by z-transforming individual metabolite concentrations, summing across metabolites, and averaging per patient. Patients with higher composite scores experienced significantly shorter survival compared with those with lower scores in both median- and quartile-based stratifications (Figure 2B, 2C). Associations remained significant after adjustment for clinical covariates, underscoring the robustness of the composite score as a predictor of outcome.

## DISCUSSION

Malignant mesothelioma remains a cancer with few reliable biomarkers for early detection or prognosis. Current blood-based assays such as MESOMARK, fibulin-3, and osteopontin have provided insights but are limited by low sensitivity, preanalytical instability, and inconsistent reproducibility (2–4). Our study demonstrates that four urinary metabolites—creatine riboside (CR), N-acetylneuraminic acid (NANA), cortisol sulfate (CS), and cholestane pentol (CP)—are significantly associated with poor-prognostic molecular signatures and adverse clinical outcomes, highlighting their potential as complementary biomarkers.

We observed that higher urinary metabolite concentrations correlated with a validated 48-gene prognostic expression signature.^7^ Although this signature is enriched for cell cycle and DNA replication pathways, the metabolites represent diverse biochemical processes, including the urea cycle, sialic acid metabolism, and steroid and cholesterol pathways.^8, 9^ Glucocorticoids and cholesterol derivatives have been shown to promote proliferation and immune modulation in cancer,^10^ suggesting that metabolite elevation may reflect systemic manifestations of tumordriven metabolic reprogramming.

Survival analyses revealed a graded, dose-dependent association: patients with three or more high metabolites had the poorest outcomes, while those with few or none had comparatively favorable survival. Importantly, metabolite levels were independent of age, sex, and disease site, supporting their robustness as biomarkers not confounded by clinical heterogeneity. A composite metabolite scores further enhanced prognostic discrimination, providing a scalable approach for clinical use.

Clinically, urinary biomarkers offer advantages over blood-based tests. Urine collection is noninvasive, inexpensive, and repeatable, allowing longitudinal monitoring in both practice and trial settings. Integration of urinary metabolite profiling with existing genomic and proteomic markers could enable more precise risk stratification and patient selection for novel therapies. Such an approach may be especially valuable in mesothelioma, where invasive sampling is often not feasible.

This study has limitations. It was based on a single cohort, and validation in larger, independent populations is required. Mechanistic links between metabolite dysregulation and mesothelioma biology remain to be elucidated, and standardized, clinically deployable assays will be needed for translation. Nevertheless, our findings establish urinary metabolites as promising prognostic biomarkers that complement existing assays and may ultimately improve patient management and clinical trial design in mesothelioma.

## Supplementary Material

Supplement 1

## Figures and Tables

**Table 1.** Mesothelioma patient characteristics

Patient characteristics	n (%)

**Total number of patients**	95
Gender	
Male	51 (54)
Female	44 (46)
Age at diagnosis, years	
<60	59 (62)
≥60	35 (37)
Unknown	1 (1)
Asbestos exposure*	
Yes	52 (55)
No	19 (20)
Unknown	24 (25)
Mesothelioma site	
Pleura	47 (49)
Peritoneum	44 (46)
Bi-Compartmental	3 (3)
Tunica Vaginalis	1 (1)
Smoking	
No	45 (47)
Yes	47 (50)
Unknown	3 (3)

* Asbestos exposure by self-report.

## Data Availability

The dataset used in this study will be made available to qualified researchers upon reasonable requests to the corresponding author, subject to institutional data use agreements and ethical approvals.

## References

[1] Janes SM, Alrifai D, Fennell DA. Perspectives on the Treatment of Malignant Pleural Mesothelioma. New England Journal of Medicine 2021;385: 1207–18. 34551230 10.1056/NEJMra1912719

[2] Robinson BWS, Creaney J, Lake R, Nowak A, Musk AW, de Klerk N, Winzell P, Hellstrom KE, Hellstrom I. Mesothelin-family proteins and diagnosis of mesothelioma. The Lancet 2003;362: 1612–6. 10.1016/S0140-6736(03)14794-0 14630441

[3] Pass HI, Levin SM, Harbut MR, Melamed J, Chiriboga L, Donington J, Huflejt M, Carbone M, Chia D, Goodglick L, Goodman GE, Thornquist MD, Fibulin-3 as a Blood and Effusion Biomarker for Pleural Mesothelioma. New England Journal of Medicine 2012;367: 1417–27. 23050525 10.1056/NEJMoa1115050 PMC3761217

[4] Pass HI, Lott D, Lonardo F, Harbut M, Liu Z, Tang N, Carbone M, Webb C, Wali A. Asbestos Exposure, Pleural Mesothelioma, and Serum Osteopontin Levels. New England Journal of Medicine 2005;353: 1564–73. 16221779 10.1056/NEJMoa051185

[5] Mathé EA, Patterson AD, Haznadar M, Manna SK, Krausz KW, Bowman ED, Shields PG, Idle JR, Smith PB, Anami K, Kazandjian DG, Hatzakis E, Noninvasive Urinary Metabolomic Profiling Identifies Diagnostic and Prognostic Markers in Lung Cancer. Cancer Research 2014;74: 3259–70. 24736543 10.1158/0008-5472.CAN-14-0109 PMC4100625

[6] Haznadar M, Diehl CM, Parker AL, Krausz KW, Bowman ED, Rabibhadana S, Forgues M, Bhudhisawasdi V, Gonzalez FJ, Mahidol C, Budhu A, Wang XW, Urinary Metabolites Diagnostic and Prognostic of Intrahepatic Cholangiocarcinoma. Cancer Epidemiology, Biomarkers & Prevention 2019;28: 1704–11. 10.1158/1055-9965.EPI-19-0453 PMC6774833 31358519

[7] Nair NU, Jiang Q, Wei JS, Misra VA, Morrow B, Kesserwan C, Hermida LC, Lee JS, Mian I, Zhang J, Lebensohn A, Miettinen M, Genomic and transcriptomic analyses identify a prognostic gene signature and predict response to therapy in pleural and peritoneal mesothelioma. Cell Reports Medicine 2023;4: 100938. 36773602 10.1016/j.xcrm.2023.100938 PMC9975319

[8] Parker AL, Toulabi L, Oike T, Kanke Y, Patel D, Tada T, Taylor S, Beck JA, Bowman E, Reyzer ML, Butcher D, Kuhn S, Creatine riboside is a cancer cell–derived metabolite associated with arginine auxotrophy. The Journal of Clinical Investigation 2022;132. 10.1172/JCI157410 PMC9282934 35838048

[9] Dobie C, Skropeta D. Insights into the role of sialylation in cancer progression and metastasis. British Journal of Cancer 2021;124: 76–90. 33144696 10.1038/s41416-020-01126-7 PMC7782833

[10] Taves MD, Otsuka S, Taylor MA, Donahue KM, Meyer TJ, Cam MC, Ashwell JD. Tumors produce glucocorticoids by metabolite recycling, not synthesis, and activate Tregs to promote growth. The Journal of Clinical Investigation 2023;133. 10.1172/JCI164599 PMC10503810 37471141

