# Supplementary material for: Urinary Metabolites and Survival in Malignant Mesothelioma
Source: medRxiv. 2025 Sep 25:2025.09.23.25336488. Preprint. [Version 1] doi: 10.1101/2025.09.23.25336488 (PMC12747252; doi:10.1101/2025.09.23.25336488)
Supplement: Supplement 1 [file NIHPP2025.09.23.25336488v1-supplement-1.pdf]

medRxiv preprint doi: https://doi.org/10.1101/2025.09.23.25336488; this version posted September 25, 2025. The copyright holder for this preprint (which was not certified by peer review) is the author/funder, who has granted medRxiv a license to display the preprint in perpetuity.

It is made available under a CC-BY 4.0 International license .

Figure S1

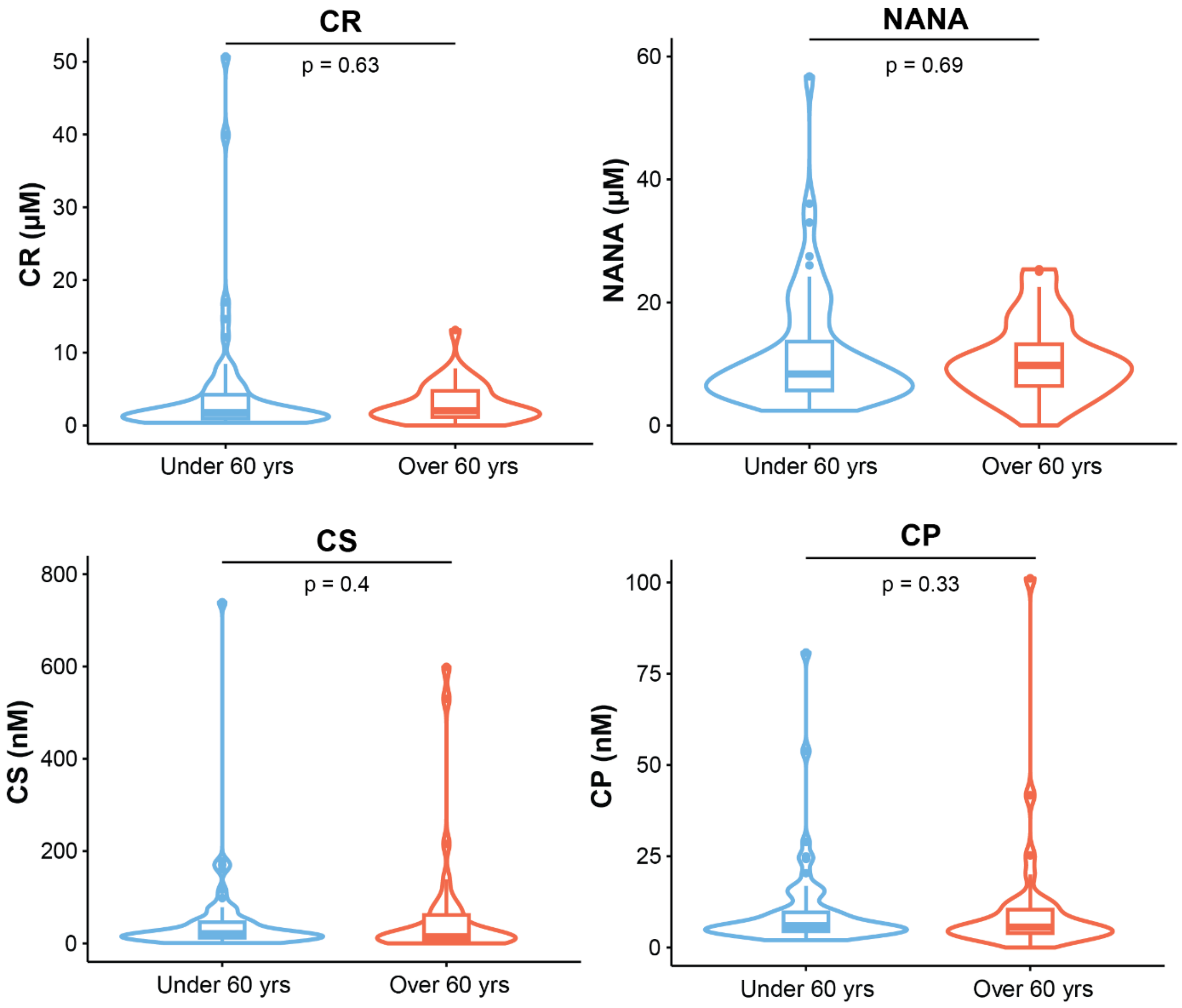

Figure S1. Metabolite concentration is unaffected by age. CR, creatine riboside (under 60 n = 59, over 60 n = 35); NANA, *N*-acetylneuraminic acid (under 60 n = 59, over 60 n = 35); CS, cortisol sulfate (under 60 n = 59, over 60 n = 33); CR, cholestane pentol (under 60 n = 59, over 60 n = 34).

medRxiv preprint doi: https://doi.org/10.1101/2025.09.23.25336488; this version posted September 25, 2025. The copyright holder for this preprint (which was not certified by peer review) is the author/funder, who has granted medRxiv a license to display the preprint in perpetuity.

It is made available under a CC-BY 4.0 International license .

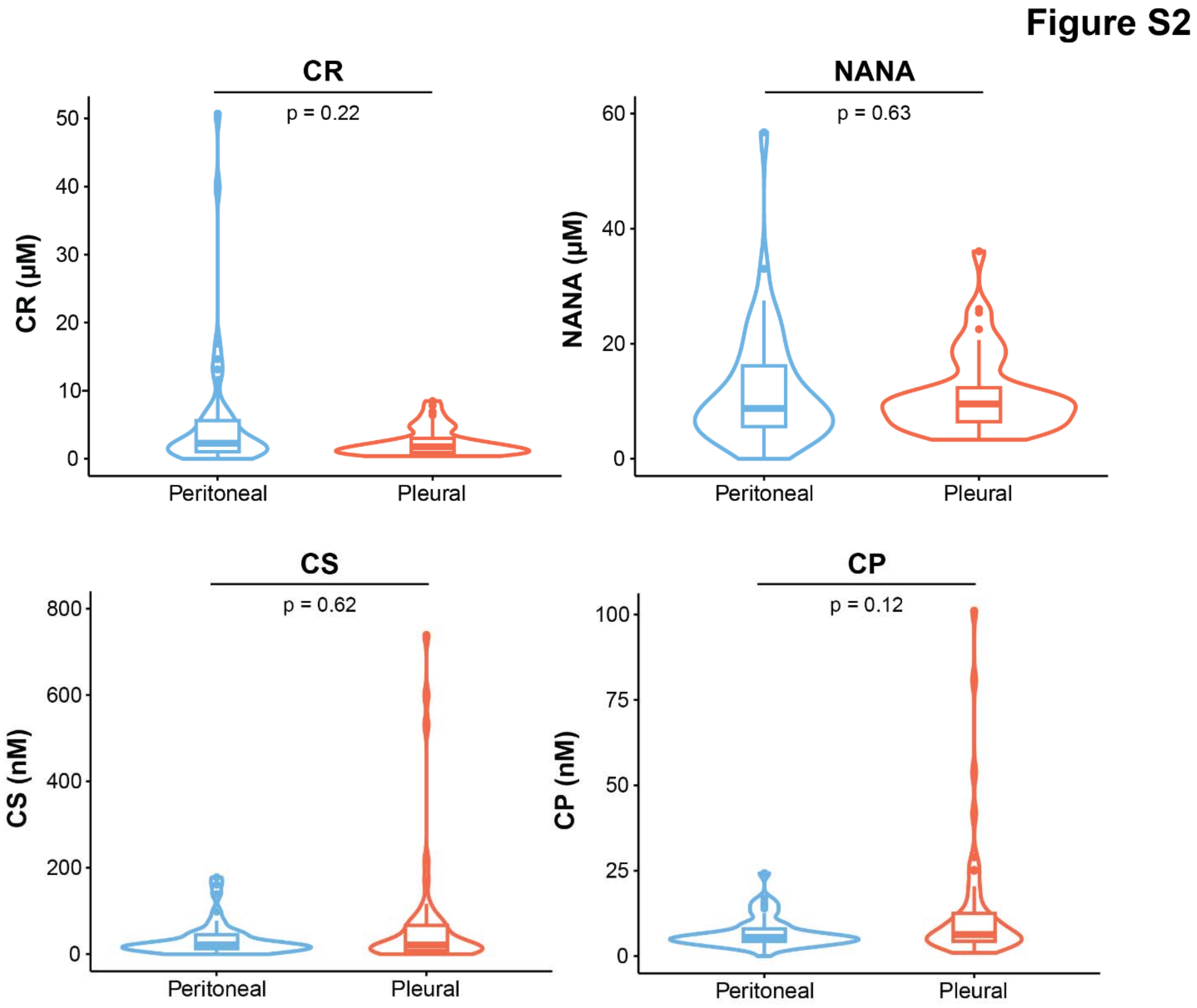

Figure S2. Metabolite concentration is unaffected by the site of disease. CR, creatine riboside (peritoneal n = 44, pleural n = 47); NANA, *N*-acetylneuraminic acid (peritoneal n = 44, pleural n = 47); CS, cortisol sulfate (peritoneal n = 43, pleural n = 46); CR, cholestane pentol (peritoneal n = 43, pleural n = 47).

medRxiv preprint doi: https://doi.org/10.1101/2025.09.23.25336488; this version posted September 25, 2025. The copyright holder for this preprint (which was not certified by peer review) is the author/funder, who has granted medRxiv a license to display the preprint in perpetuity.

It is made available under a CC-BY 4.0 International license .

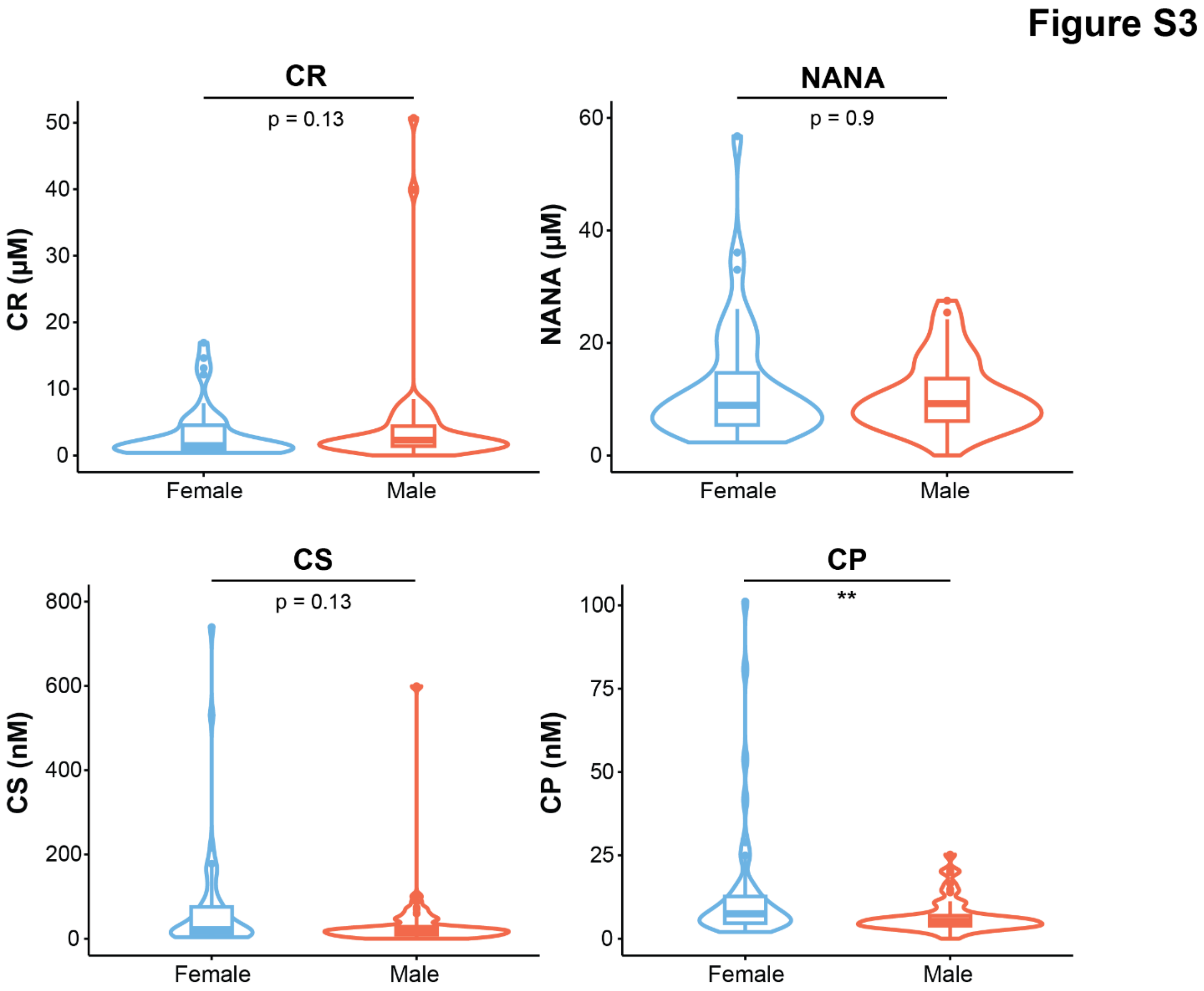

Figure S3. Metabolite concentration is largely unaffected by gender. CR, creatine riboside (female n = 44, male n = 51); NANA, *N*-acetylneuraminic acid (female n = 44, male n = 51); CS, cortisol sulfate (female n = 44, male n = 49); CR, cholestane pentol (female n = 44, male n = 50). ** $p < 0.01$, ** $p < 0.01$.
